# Supplementary material for: Structure-based design of a soluble human cytomegalovirus glycoprotein B antigen stabilized in a prefusion-like conformation
Source: Proc Natl Acad Sci U S A. 2024 Sep 4;121(37):e2404250121. doi: 10.1073/pnas.2404250121 (PMC11406251; doi:10.1073/pnas.2404250121)
Supplement: Supplementary file 1 — Appendix 01 (PDF) [file pnas.2404250121.sapp.pdf]

PNAS

Supporting Information for

Structure-based design of a soluble human cytomegalovirus glycoprotein B antigen stabilized in a prefusion-like conformation

Authors: Madeline R. Sponholtz †, Patrick O. Byrne †, Alison G. Lee, Ajit R. Ramamohan, Jory A. Goldsmith, Ryan S. McCool, Ling Zhou, Nicole V. Johnson, Ching-Lin Hsieh, Megan Connors, Krithika Karthigeyan, Chelsea M. Crooks, Adelaide S. Fuller, John D. Campbell, Sallie R. Permar, Jennifer A. Maynard, Dong Yu, Matthew J. Bottomley, Jason S. McLellan

Corresponding author: Jason S. McLellan
Email: jmclellan@austin.utexas.edu

This PDF file includes:

Supporting Text
Figures S1 to S15
Tables S1 to S3
SI References

Supporting Text

Extended Methods

Production of HCMV gB protein for cryo-EM. HEK293F cells were transiently transfected with plasmids encoding either HCMV gB Base or gB-C7 using PEI, with 5 µM kifunensine added approximately 4 hours after transfection. After 5–6 days, medium was harvested by centrifugation and 0.22 µm filtered. Secreted proteins were concentrated and exchanged into PBS using a tangential flow filtration cassette (PALL), then purified by Strep-Tactin Sepharose resin (IBA Lifesciences) and concentrated as described above for purification of HCMV gB variants. For gB-C7, eluate was then treated with 5% (w/w) HRV3C protease at 4 °C overnight to remove affinity tags. For gB Base, eluate was flash-frozen in liquid nitrogen. Samples of gB Base were thawed in a room temperature water bath just before injection onto a HiLoad Superose 6 16/70 pg column (Cytiva). For gB-C7, solution was injected onto a HiLoad Superose 6 16/70 pg column (Cytivia) following HRV3C digestion. The running buffer was composed of 2 mM Tris pH 8.0, 200 mM NaCl, and 0.02% (w/v) sodium azide. Desired fractions were pooled, concentrated, aliquoted and flash-frozen in liquid nitrogen for further analysis.

Expression and purification of Fabs for structural and biophysical studies. For 1G2 and 7H3, a stop codon was introduced before the hinge region of the heavy chains to generate fragments of antigen binding (Fabs). HEK293F cell cultures were transiently transfected with plasmids encoding 1G2 and 7H3 heavy and light chains using PEI. Cultures were harvested 6 days after transfection and the medium was separated from the cells by centrifugation. Supernatant was passed through a 0.22 µm filter and then concentrated using a tangential flow filtration cassette (PALL) before Fab was purified with CaptureSelect™ IgG-CH1 Affinity Matrix (ThermoFisher). Bound Fab was eluted in a buffer containing 100 mM glycine pH 3.0. The protein elution was immediately neutralized with 1 M Tris pH 8.0, and then further purified by size-exclusion chromatography using a HiLoad 16/600 Superdex 200 pg column (GE Healthcare) into SEC running buffer. All protein samples were concentrated using Amicon Ultra centrifugal filter units (MilliporeSigma) to between 1 and 10 mg/ml, then flash-frozen in liquid nitrogen and stored at −80 °C.

Differential scanning fluorimetry. In white-walled 96-well PCR plates (VWR), solutions were prepared with a final concentration of 15X SYPRO Orange (ThermoFisher Scientific, 5,000X SYPRO Orange Protein Gel Stain in DMSO) and 0.45 mg/mL purified HCMV gB samples. The absorbance of the working SYPRO Orange solution was 0.295 at 468 nm (1 mm path length in a NanoDrop One, ThermoFisher Scientific). Plates were sealed and loaded into a LightCycler 480 differential scanning fluorimeter (Roche) equipped with a 100W xenon excitation lamp. We used a 465 nm excitation filter (25 nm half band width) and a 580 nm emission filter (20 nm half band width). The fluorescence in each well was monitored as the temperature of the plate was increased from 25 °C to 90 °C. Data were plotted as the change in fluorescence with respect to temperature (dF/dT) as a function of temperature.

Immunized serum ELISAs. SM5-1 antibody with murine IgG2a Fc (SM5-1 mFc) was produced in ExpiCHO cells via transient transfection and purified from cell culture supernatant with Protein A resin for use as an ELISA standard. ELISAs were performed at room temperature using PBS + 0.1% (w/v) Tween 20 (PBST) as the wash buffer and 5% milk in PBST as the blocking buffer. All wells were washed three times with 300 µL PBST following each step. High-binding 96-well plates were coated with gB Base or gB-C7 protein in Dulbecco's PBS at a concentration of 1 µg/mL for 1 hour. Plates were then blocked with blocking buffer for 30 minutes. The standard (5 µg/mL SM5-1 mFc mixed with 1:100 unimmunized mouse serum) and 1:100 diluted immunized mouse sera were serial diluted by a factor of √10 in blocking buffer and applied to the plate for 1 hour. Next, goat anti-mouse Ig HRP (Southern Biotech, Cat. #1010-05) secondary antibody was diluted 1:2000 in blocking buffer and applied to the plate for 1 hour. Plates were then developed using 3,3′,5,5′-tetramethylbenzidine (TMB) substrate solution and quenched with 1 N HCl. Absorbance at 450 nm was recorded using a SpectraMax M5 plate reader (Molecular Devices). ELISA curves were fit to a 4-parameter logistic (4PL) curve using Microsoft Excel and normalized to the standard of their respective plates. The concentrations of antibodies in immunized mouse sera relative to the standard were estimated by fitting 4PL curves of serum samples to the standard curve. The distribution of

EC50 data was determined to be log-normal by Shapiro-Wilk test for normality. Antibody titers are shown as averages of two technical replicates from two separate experimental replicates.

Binding antibody multiplex assay (BAMA)

A Luminex-based binding antibody multiplex assay (BAMA) was used to measure IgG binding to select gB antigenic domains (90). A portion of HCMV gB DIV corresponding to AD-1 comprising residues 552–647 of HCMV gB Merlin Strain (UniProtKB: F5HB53) was purchased from MyBioSource (Catalog # MBS485117; San Diego, CA) and HCMV gB DI, DII, and DI+II were produced in house using the Merlin strain sequence (UniProtKB: F5HB53). DI corresponding to AD-5 comprising residues 133–343 of HCMV gB Merlin strain (UniProtKB: F5HB53) was not tagged due to hypothesized steric hindrance. Sequences encoding HCMV gB DII corresponding to AD-4 comprising residues 112–133 and 343–438 of HCMV gB Merlin strain (UniProtKB: F5HB53) and gB DI+II corresponding to AD-4+5 comprising residues 112–438 of HCMV gB Merlin strain (UniProtKB: F5HB53) were tagged at the N-terminus with the UL132 signal peptide sequence MPAPRGPLRATFLALVAFGLLLQIDLS and hemagglutinin (HA) tag, and at the C-terminus with an avidin and polyhistidine tag. The discontinuous sequence encoding gB DII was joined with the flexible linker Ile-Ala-Gly-Ser-Gly. Nucleotides were codon optimized for mammalian cells, synthesized (Genscript), then cloned into pcDNA3.1(+) mammalian expression vector (Invitrogen). Plasmids were transiently transfected into 293F suspension cells using polyethylenimine transfection reagent (Sigma-Aldrich). Supernatant was harvested 2–5 days later and purified using Ni2+-NTA resin (Thermo Fisher Scientific). Purity and identity were confirmed by Western blot using monoclonal antibody SM5-1 (DII and DI+II) and SM10 (DI). Antigens were covalently coupled to fluorescent magnetic beads (Luminex). Plasma samples were diluted either 1:50 (gB DI and gB DIV) or 1:5000 (gB DI+II and gB DII) and incubated with antigen-coupled beads. Goat Anti-Mouse IgG-Human-PE (Southern Biotech) secondary antibody was used for detection and assay plates were read on a Bio-Plex 200 machine (Bio-Rad). A serial dilution of HCMV-hyperimmunoglobulin (Cytogam), and an RSV-specific monoclonal antibody (Synagis) point dilution were included on each plate as positive and negative controls, respectively and to assess inter-plate variability. These human controls were run with a Goat Anti-Human IgG-PE secondary (Southern Biotech). Blank wells were included to determine background signal and reported MFIs are background subtracted. Dotted lines on the graph indicate average of unimmunized sera. All samples were run in duplicate. Any sample that had a CV over 25%, machine sampling errors, or bead counts below 100 were repeated. Quality control was performed using a custom R-script that determines whether QC criteria are met for each sample.

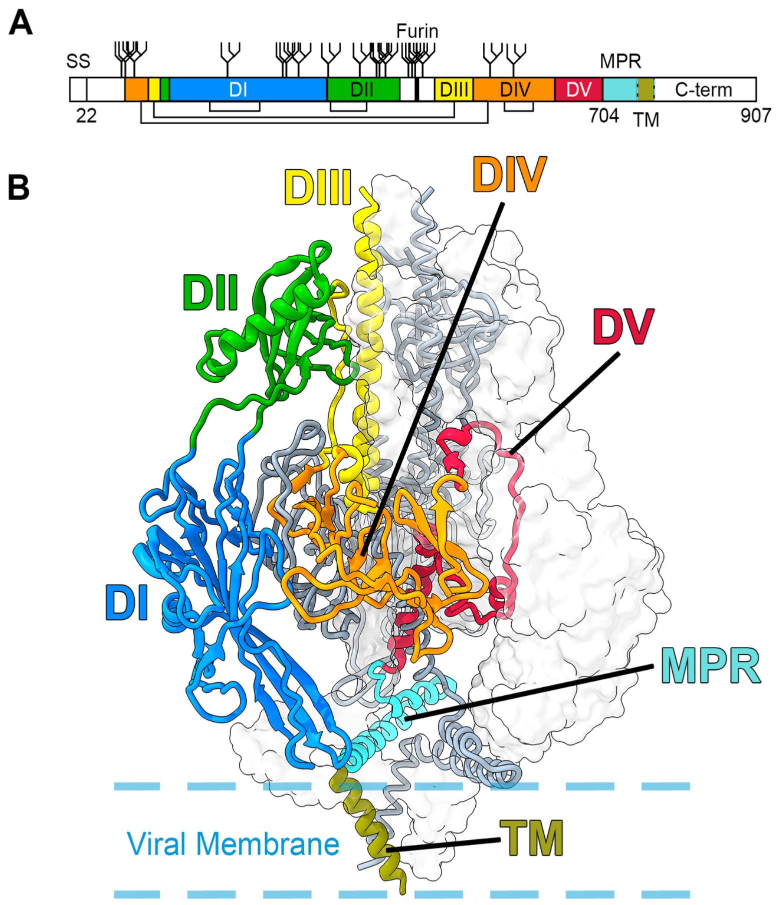

Figure S1. Domain architecture of HCMV gB. (**A**) Schematic of wild-type HCMV gB. Native disulfide bonds are shown as connected lines. N-linked glycosylation sites are shown as branched lines. The native furin cleavage site is shown as a thick black line. The N-terminal signal sequence (SS) is shown as a white box. Structural domain I (DI) is colored blue, DII is colored green, DIII is colored yellow, DIV is colored orange, DV is colored red, the membrane-proximal region (MPR) is colored cyan, the transmembrane domain (TM) is colored olive and flanked by dashed blue lines, and the C-terminal domain (C-term) is white. (**B**) Side view of trimeric prefusion HCMV gB (PDB ID: 7KDP) (1). One protomer is colored as in A and shown as a ribbon diagram, one protomer is colored gray and shown as a cartoon trace of the α-carbon backbone, and one protomer is shown as a transparent surface. The approximate location of the viral membrane is shown as dashed blue lines for orientation.

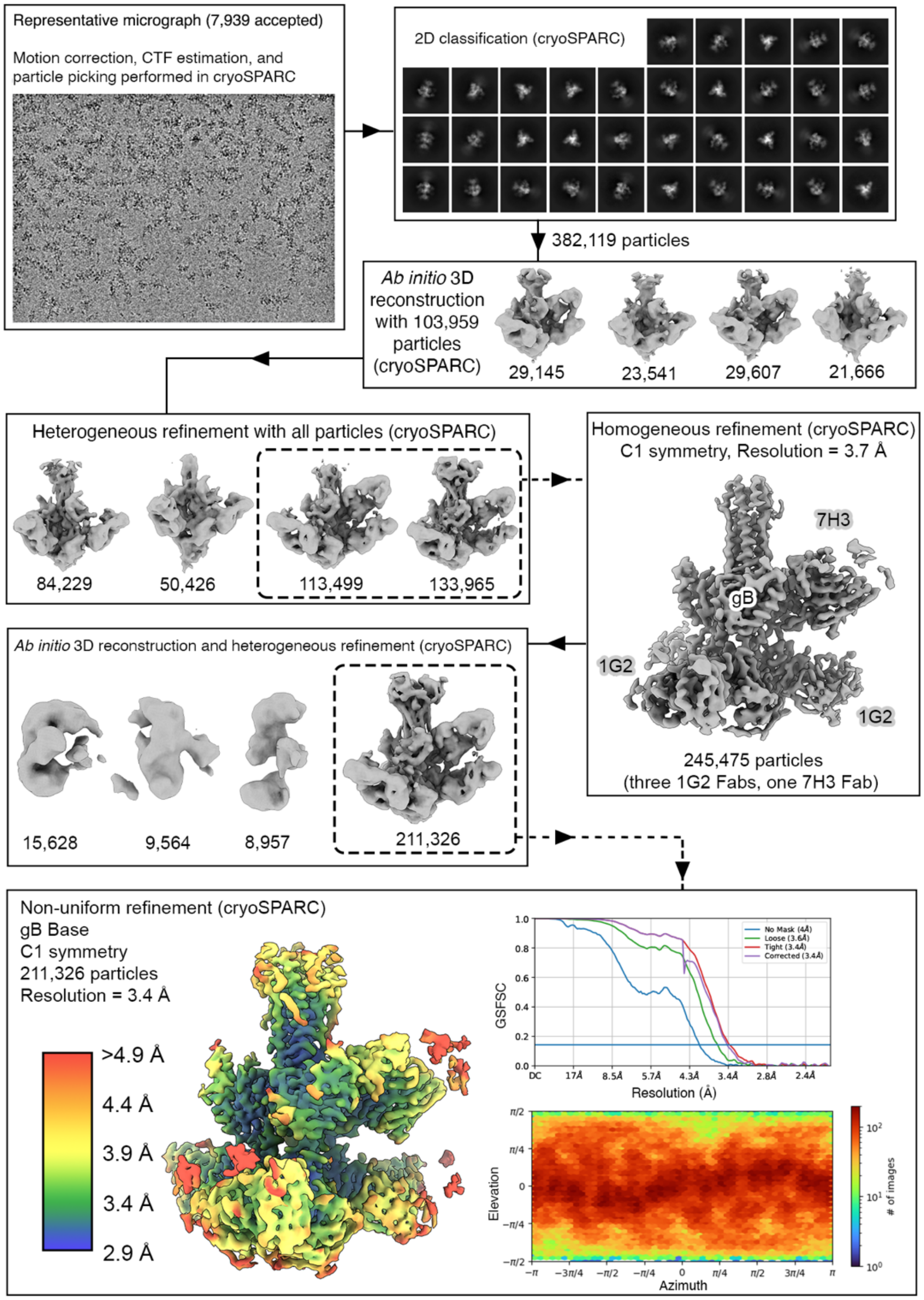

Figure S2. Cryo-EM data collection and processing summary for the structure of gB Base in complex with 1G2 and 7H3 Fabs. Cryo-EM processing workflow consisted of the following steps performed in cryoSPARC v4.2.0: motion correction, CTF estimation, particle picking, 2D classification, *ab initio* 3D reconstruction, iterative heterogenous refinement, homogenous refinement, and non-uniform refinement with C1 symmetry. The number of particles used in each step is listed. The final, highest resolution reconstruction is shown at the bottom of the figure, colored as a rainbow according to the estimated local resolution from 2.9 Å (blue) to 4.9 Å (red) resolution. Gold-standard Fourier shell correlation and directional distribution plots are shown to the right of the high-resolution reconstruction.

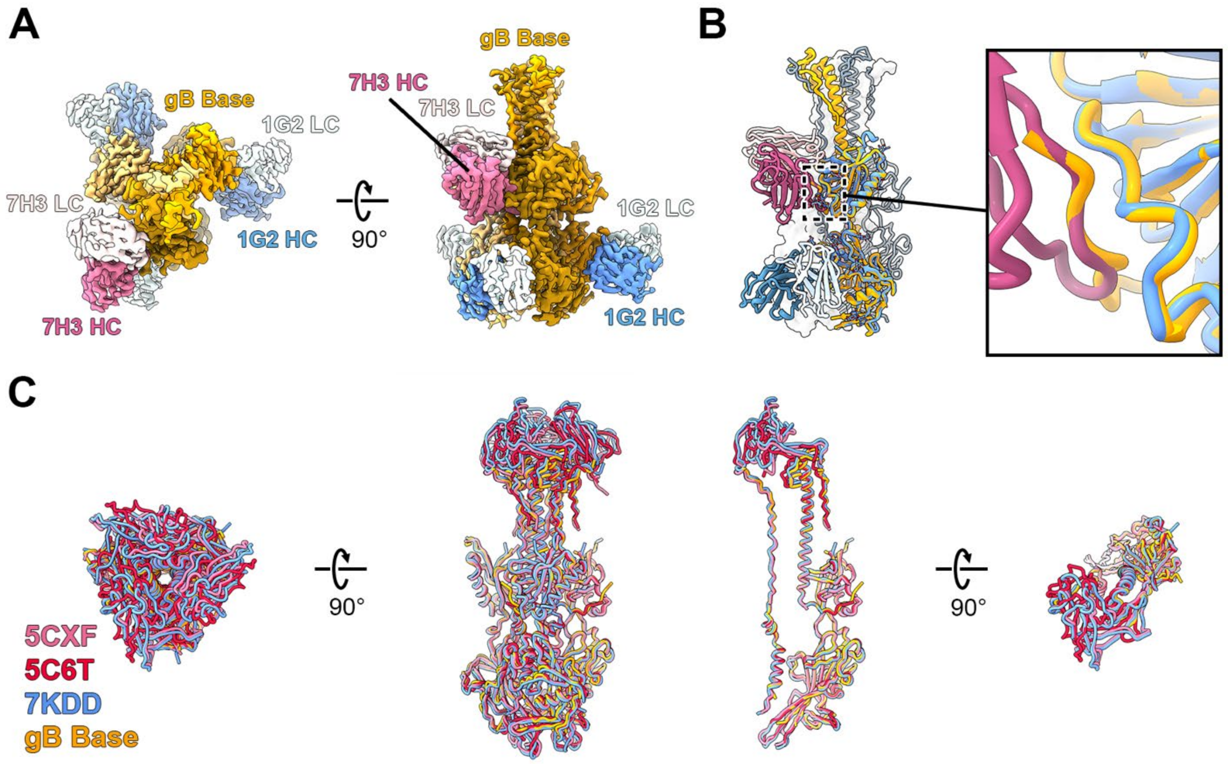

Figure S3. Cryo-EM structure of gB Base bound to 1G2 and 7H3 Fabs. (**A**) Top (*left*) and side (*right*) views of the EM map of HCMV gB ectodomain gB Base complexed with 1G2 and 7H3 Fabs. (**B**) The gB Base complex model. One protomer of the model is colored as in Figure 1A and shown as a ribbon diagram, the second is colored gray and shown as a cartoon tube trace of the α-carbon backbone, and the third is shown as a transparent surface. The structure of an unbound protomer (yellow) is shown as a ribbon diagram and superimposed with the protomer bound by 7H3 Fab. The inset shows a zoomed view of a flexible loop displaced by 7H3 Fab. (**C**) The structure of gB Base (yellow) is superimposed with the previously determined structures of postfusion HCMV gB (PDB IDs: 5CXF, 5C6T, and 7KDD in red, pink, and blue, respectively) (1-3), all shown as cartoon tube traces of the α-carbon backbones. Top and side views are shown for the superimposition of postfusion HCMV gB both as a trimer on the left and as a single protomer on the right.

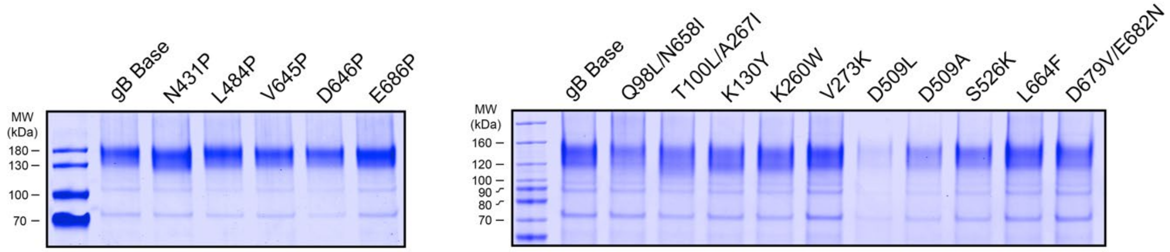

Figure S4. Reducing SDS-PAGE for single and paired substitution variants. The molecular weight ladder is in the left-most lane of each gel with standards indicated at the left in kDa.

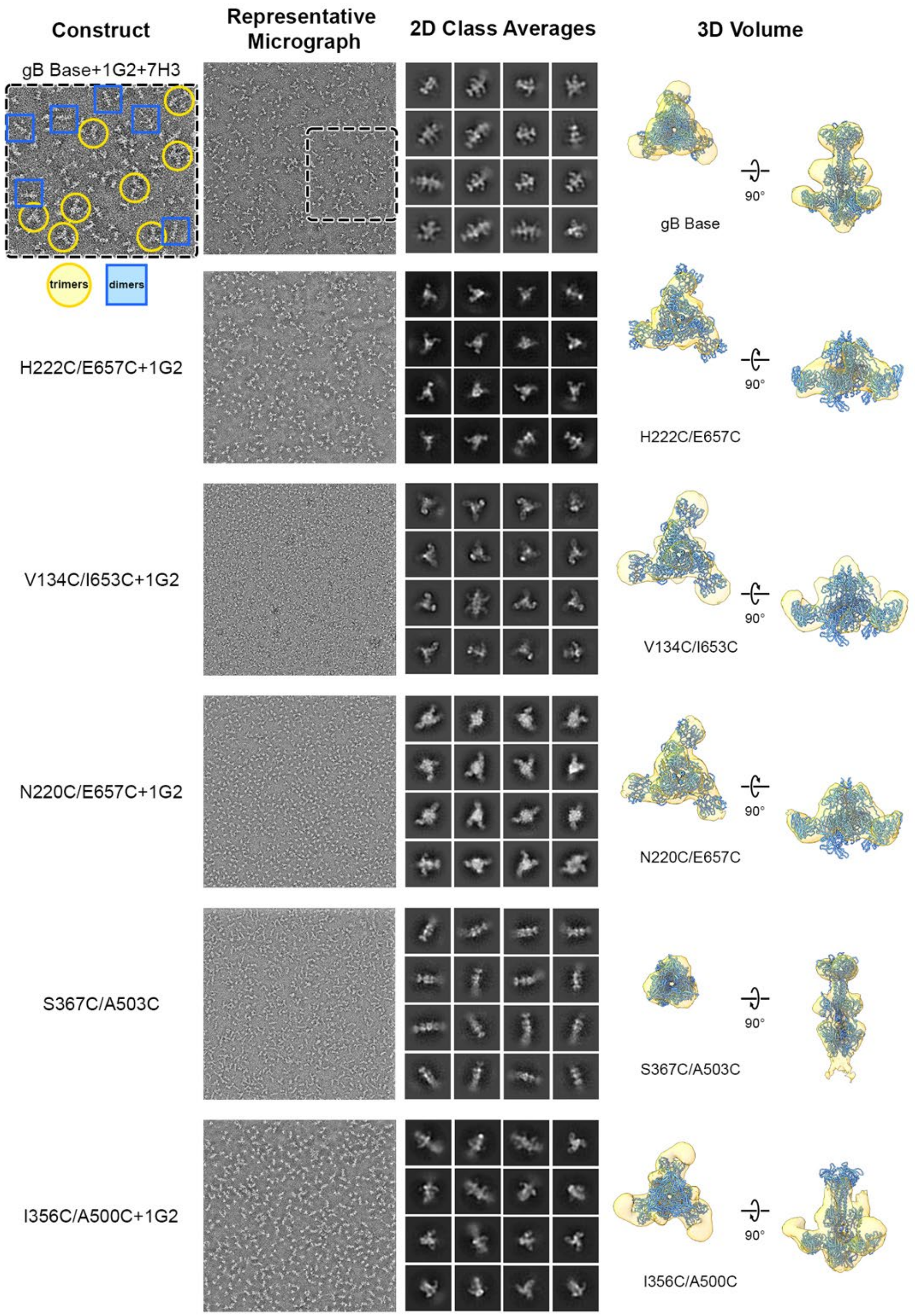

Figure S5 (continues on next page). Negative-stain EM for gB Base and select gB variants. Representative negative-stain EM (ns-EM) micrographs, 2D class averages, and low-resolution 3D reconstructions with imposed C3 symmetry for gB Base and select gB variants. The gB Base inset (*top, left*) shows a zoomed view of a representative micrograph with higher-order oligomers (rosettes) highlighted with blue squares and yellow circles, indicating dimers of gB trimer and trimers of gB trimer, respectively.

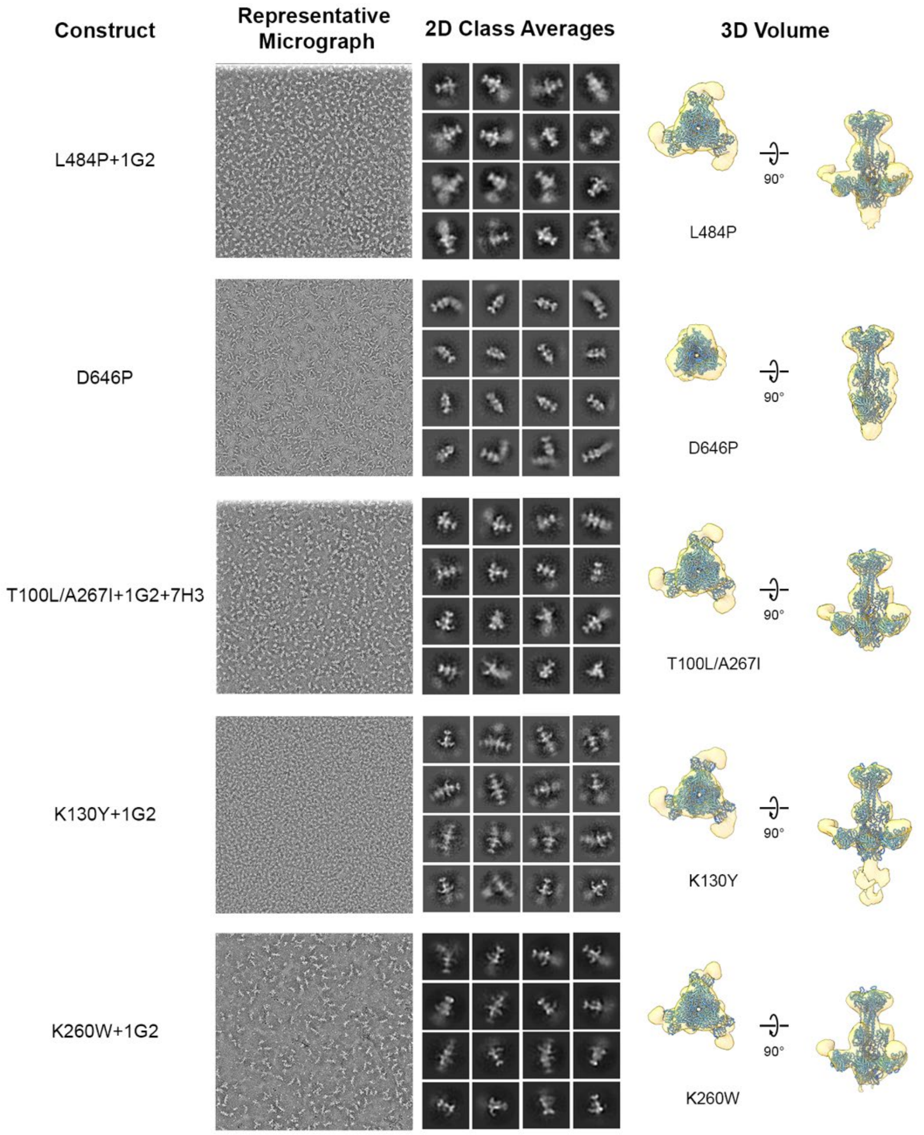

Figure S5 (continued from previous page). Negative-stain EM for gB Base and select gB variants. Representative negative-stain EM (ns-EM) micrographs, 2D class averages, and low-resolution 3D reconstructions with imposed C3 symmetry for gB Base and select gB variants.

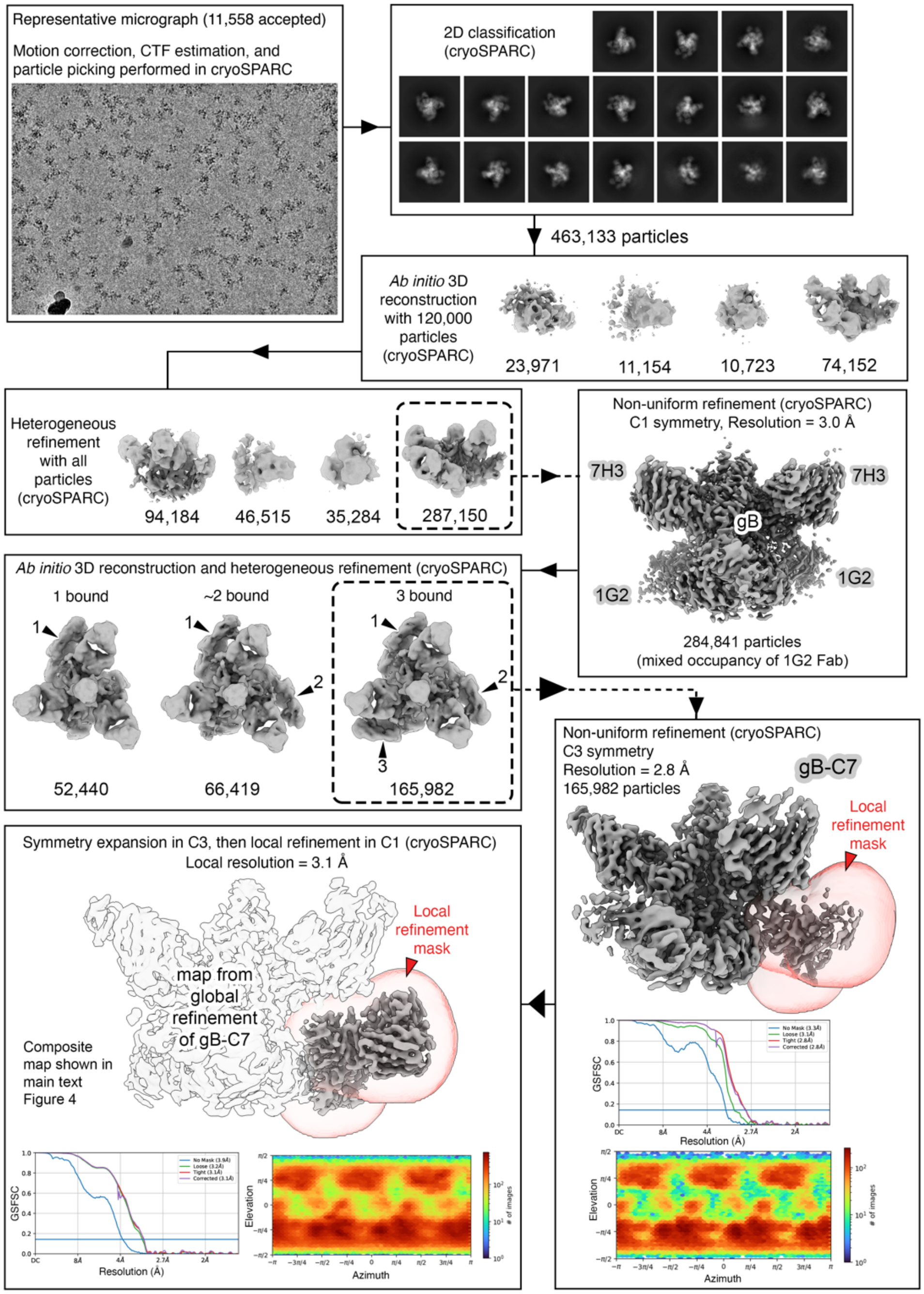

Figure S6. Cryo-EM data collection and processing summary for the structure of gB-C7 in complex with 1G2 and 7H3 Fabs. Cryo-EM processing workflow consisted of the following steps performed in cryoSPARC v4.2.0: motion correction, CTF estimation, particle picking, 2D classification, *ab initio* 3D reconstruction, iterative heterogenous refinement, homogenous refinement, and non-uniform refinement with C3 symmetry. A mask was created around domain I and 1G2 using ChimeraX and imported to cryoSPARC for local refinement with C1 symmetry using the C3 symmetry-expanded particle stack. The number of particles used in each step is listed. The final, highest-resolution reconstructions for the global and local refinements are shown at the bottom of the figure above gold-standard Fourier shell correlation and directional distribution plots.

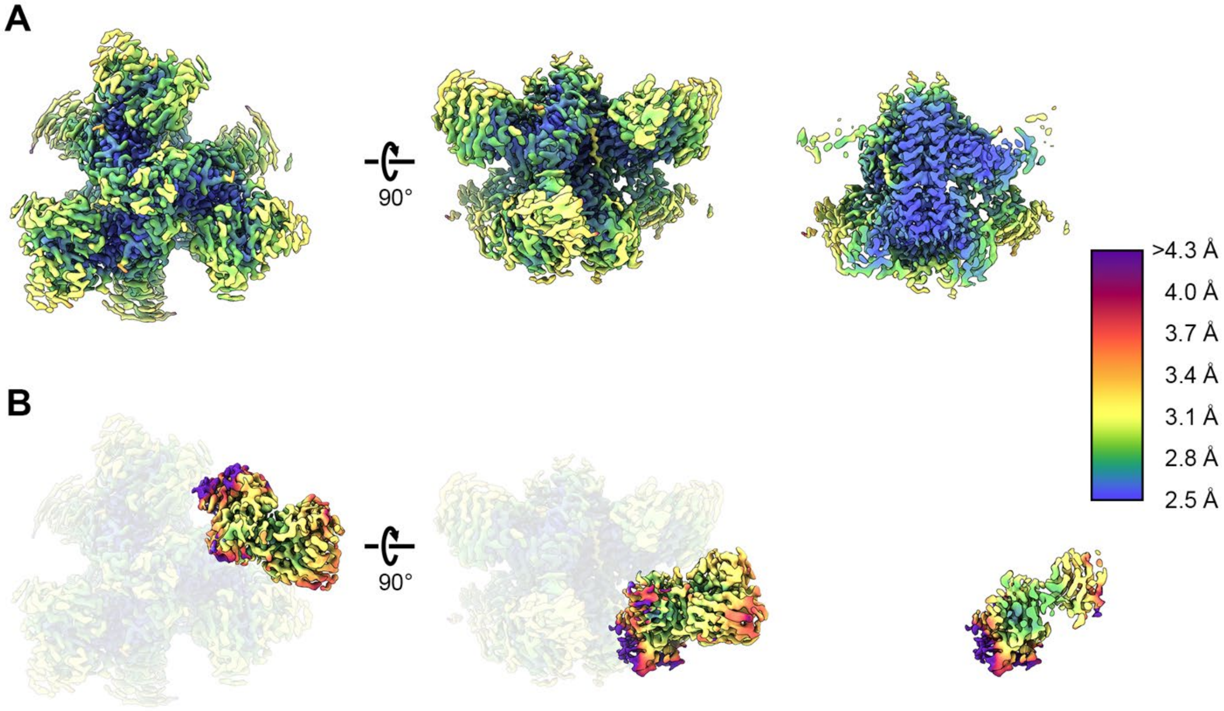

Figure S7. Cryo-EM maps colored by local resolution from global and local refinement of gB-C7. The (**A**) global and (**B**) local refinement maps of gB-C7 are colored as a rainbow according to the estimated local resolution from 2.5 Å (blue) to 4.3 Å (purple) resolution.

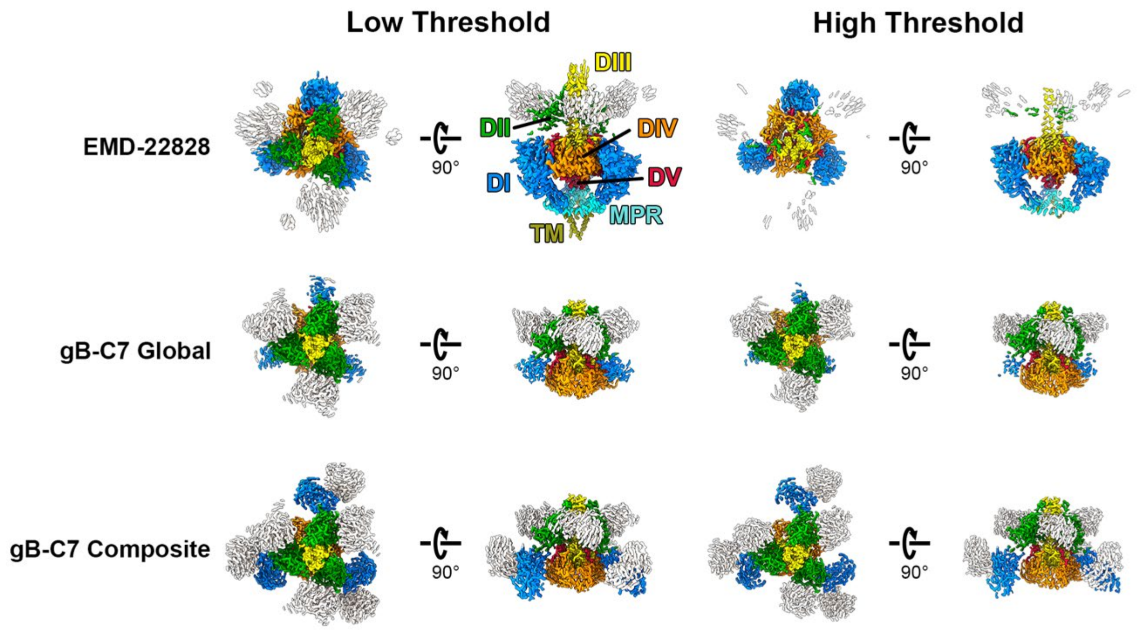

Figure S8. Cryo-EM maps at different thresholds*.* Top and side views of the previously determined EM map of prefusion HCMV gB complexed with SM5-1 Fabs (EMD-22828, PDB ID: 7KDP) (1) and the global and composite EM maps of HCMV gB-C7 complexed with 1G2 and 7H3 Fabs at lower and higher thresholds. Each map is colored based on its correspondence with structural domains of gB, with domain I (DI) colored blue, DII colored green, DIII colored yellow, DIV colored orange, DV colored red, the membrane-proximal region (MPR) colored cyan, and the transmembrane domain (TM) colored olive. Regions of the map corresponding to antibodies are white.

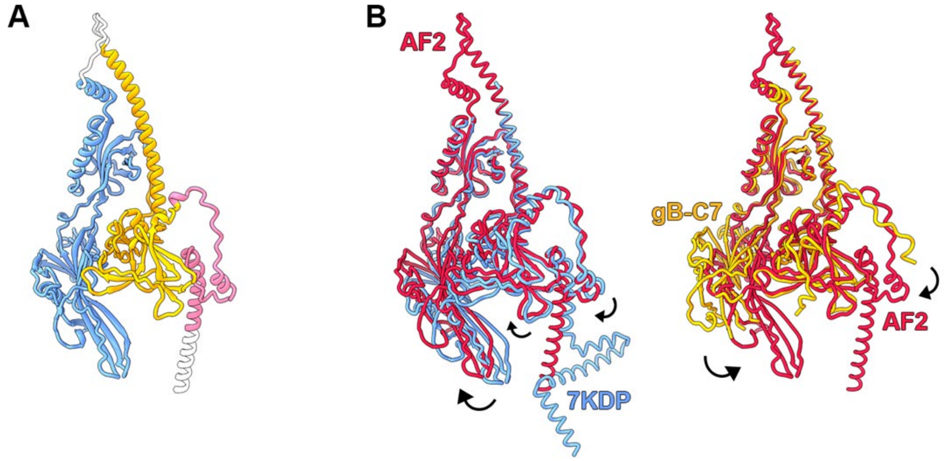

Figure S9. AlphaFold2-predicted HCMV gB protomer in the prefusion conformation. (**A**) AlphaFold2 (AF2) prefusion gB protomer model is shown as a ribbon diagram. The previously determined structure of prefusion HCMV gB (PDB ID: 7KDP) (1) was used as a template for AF2 prediction (4). The first and second regions expected to move during the conformational rearrangement from pre-to-postfusion gB are colored blue and pink, respectively, and the region that does not undergo substantial rearrangement is colored yellow. (**B**) The AF2 model (red) is superimposed with the previously determined structure of prefusion HCMV gB (blue, PDB ID: 7KDP) and the structure of gB-C7 (yellow), all shown as cartoon tube traces of the α-carbon backbones. Shifts in domain arrangement are highlighted with arrows.

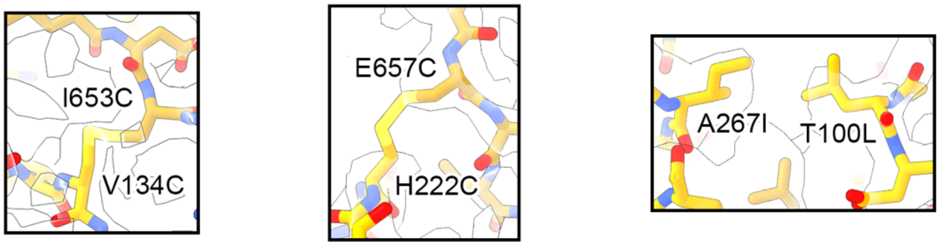

Figure S10. gB-C7 substitutions supported by the cryo-EM map. The model of HCMV gB combination variant gB-C7 is shown in gold sticks fit in the transparent composite EM map of the HCMV gB-C7 complex. Panels show close views of the substitutions that comprise design gB-C7: (**A**) V134C/I653C, (**B**) H222C/E657C, and (**C**) T100L/A267**I**. Sulfur atoms are shown in yellow, nitrogen atoms in blue, and oxygen atoms in red.

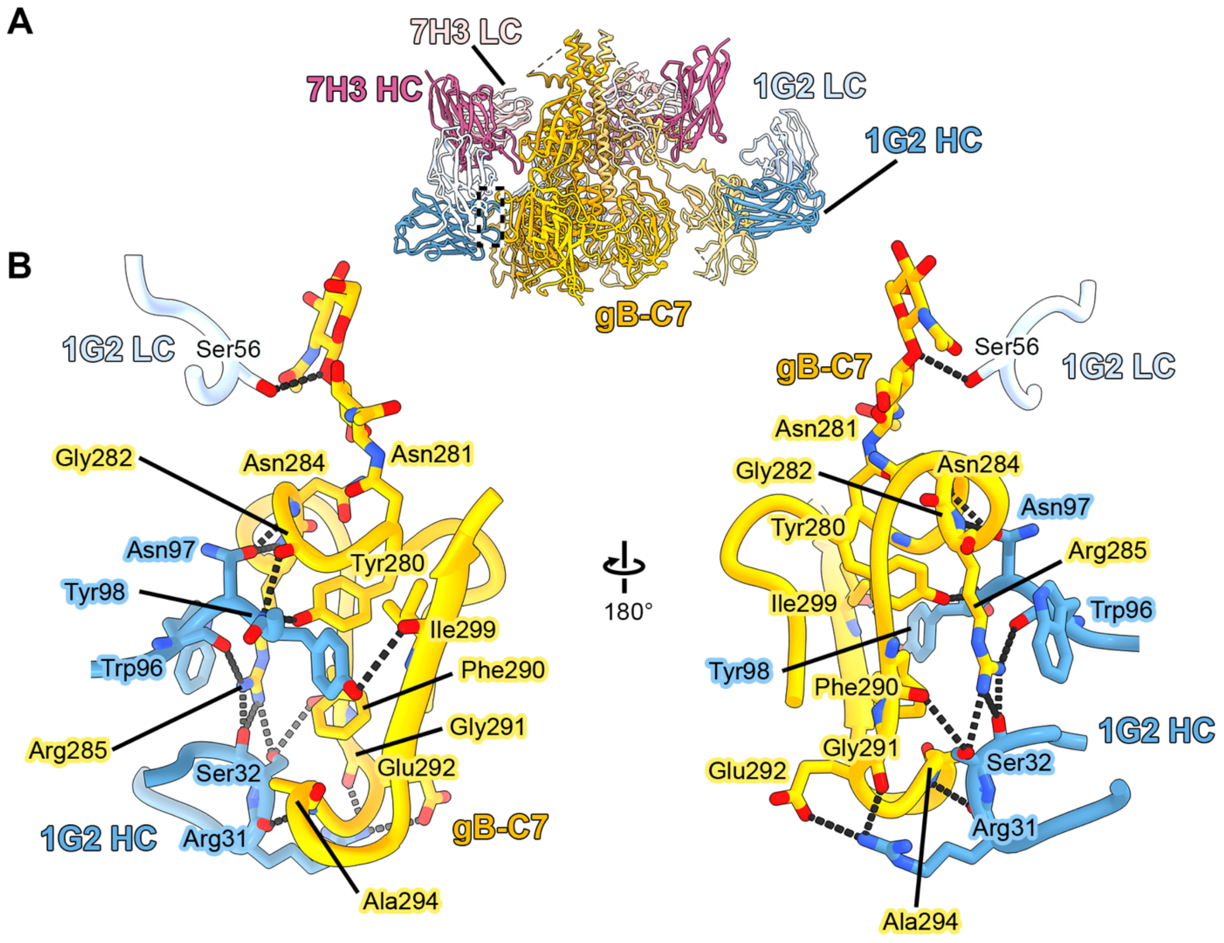

Figure S11. The gB-C7:1G2 Fab interface. (**A**) Model of gB-C7 (shades of yellow colored by protomer) complexed with 1G2 (heavy chain: blue, light chain: light blue) and 7H3 (heavy chain: pink, light chain: light pink) Fabs, with the region shown in B highlighted with dashed lines. (**B**) Zoomed view of the binding interface between 1G2 and gB-C7; the interface comprises 13 hydrogen bonds and 663 Å^2 of buried surface area on gB-C7. The gB-C7:1G2 interface is highly similar to the previously described postfusion gB:1G2 interface (PDB ID: 5C6T) (2) with an RMSD of 0.4 Å across the 21 Cα atoms comprising the continuous 1G2 epitope on gB-C7. Hydrogen bonds are shown as black dashes with interacting residues labeled and shown as sticks. Sulfur atoms are shown in yellow, nitrogen atoms in blue, and oxygen atoms in red.

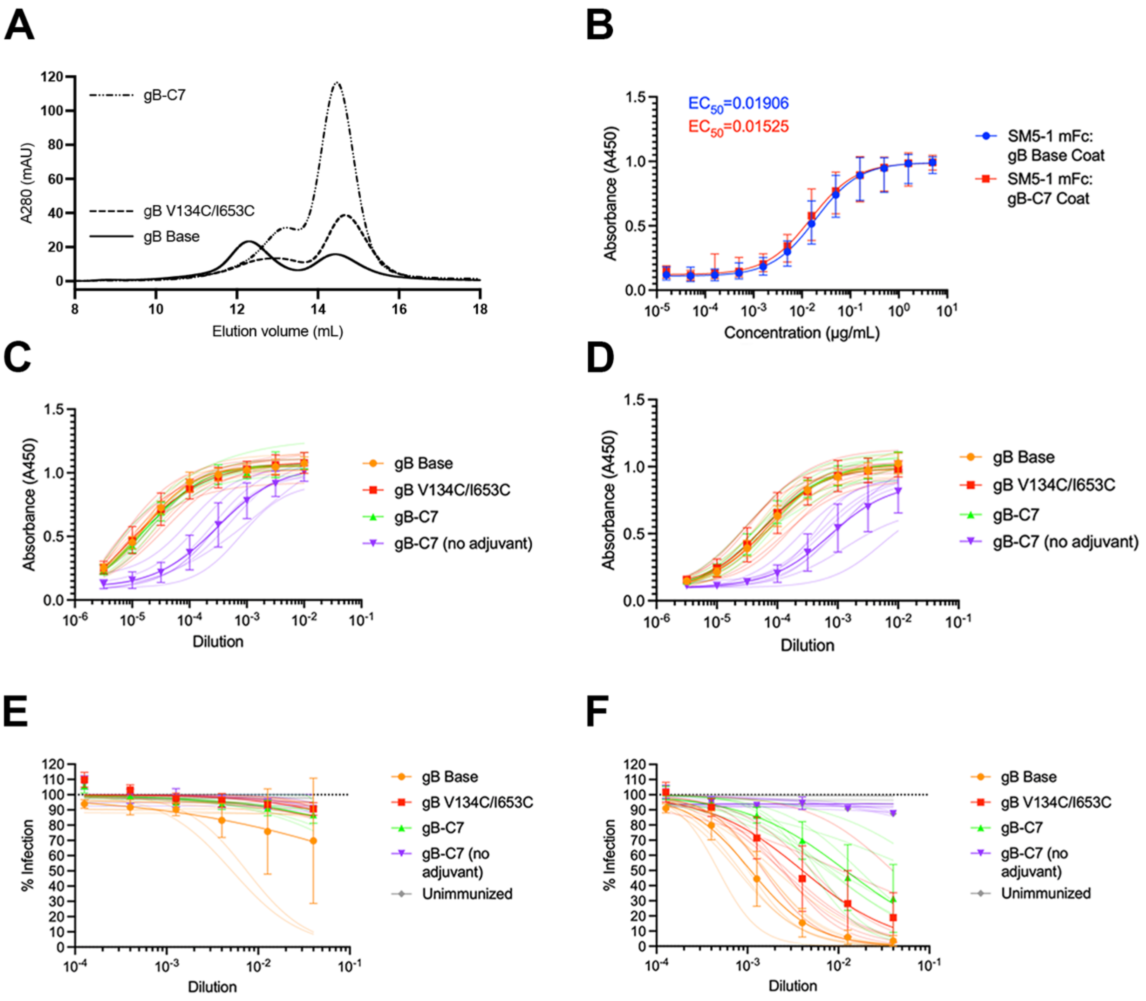

Figure S12. Individual ELISA and neutralization curves for anti-gB mouse sera. ELISA plates were coated with gB Base or gB-C7 and incubated with the SM5-1 murine IgG2a Fc (mFc) control or mouse serum. Bound mouse antibodies were detected using goat anti-mouse Ig HRP. (**A**) Size-exclusion chromatography (SEC) traces of purified gB constructs gB Base, gB V134C/I653C, and gB-C7 prepared for immunization. (**B**) SM5-1 mFc binds with similar affinity to gB Base and gB-C7 on ELISA. Plotted curves and error bars represent the average and standard deviation across 33 ELISA plates. Sera samples were diluted by √10 from 1 x 10^{-2}–3.16 x 10^{-6} and incubated on ELISA plates coated with (**C**) gB Base or (**D**) gB-C7. Immunized mouse serum was incubated with AD169-GFP in a √10-fold dilution series from 4 x 10^{-2}–1.3 x 10^{-4} (**E**) without or (**F**) with 12.5% guinea pig complement prior to incubation with human MRC-5 fibroblasts. Individual mice are represented by lighter-colored lines, which are averages across two independent experiments. The averages of each group are shown in heavier lines with error bars representing the standard deviation across each group (*n* = 8 mice/group).

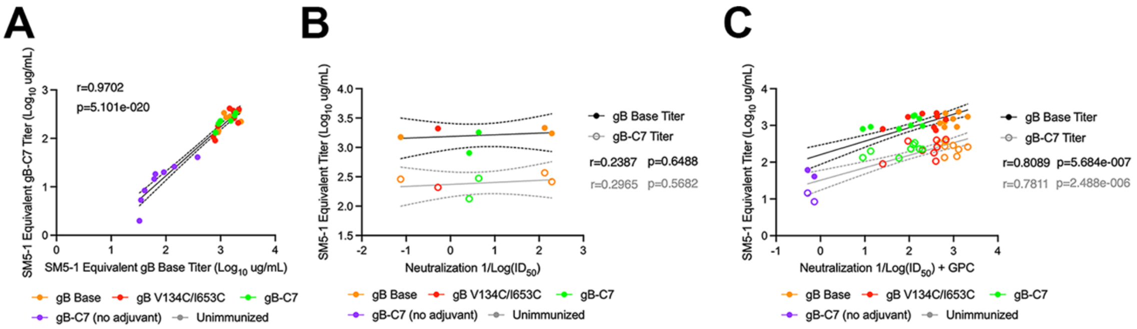

Figure S13. Correlations between gB binding and neutralizing antibody titers. Pearson's correlation coefficients (r) and p-values were calculated between (**A**) gB Base binding and gB-C7 binding antibody titers, (**B**) neutralization and gB Base binding or gB-C7 binding antibody titers, and (**C**) neutralization in the presence of 12.5% guinea pig complement and gB Base binding or gB-C7 binding antibody titers.

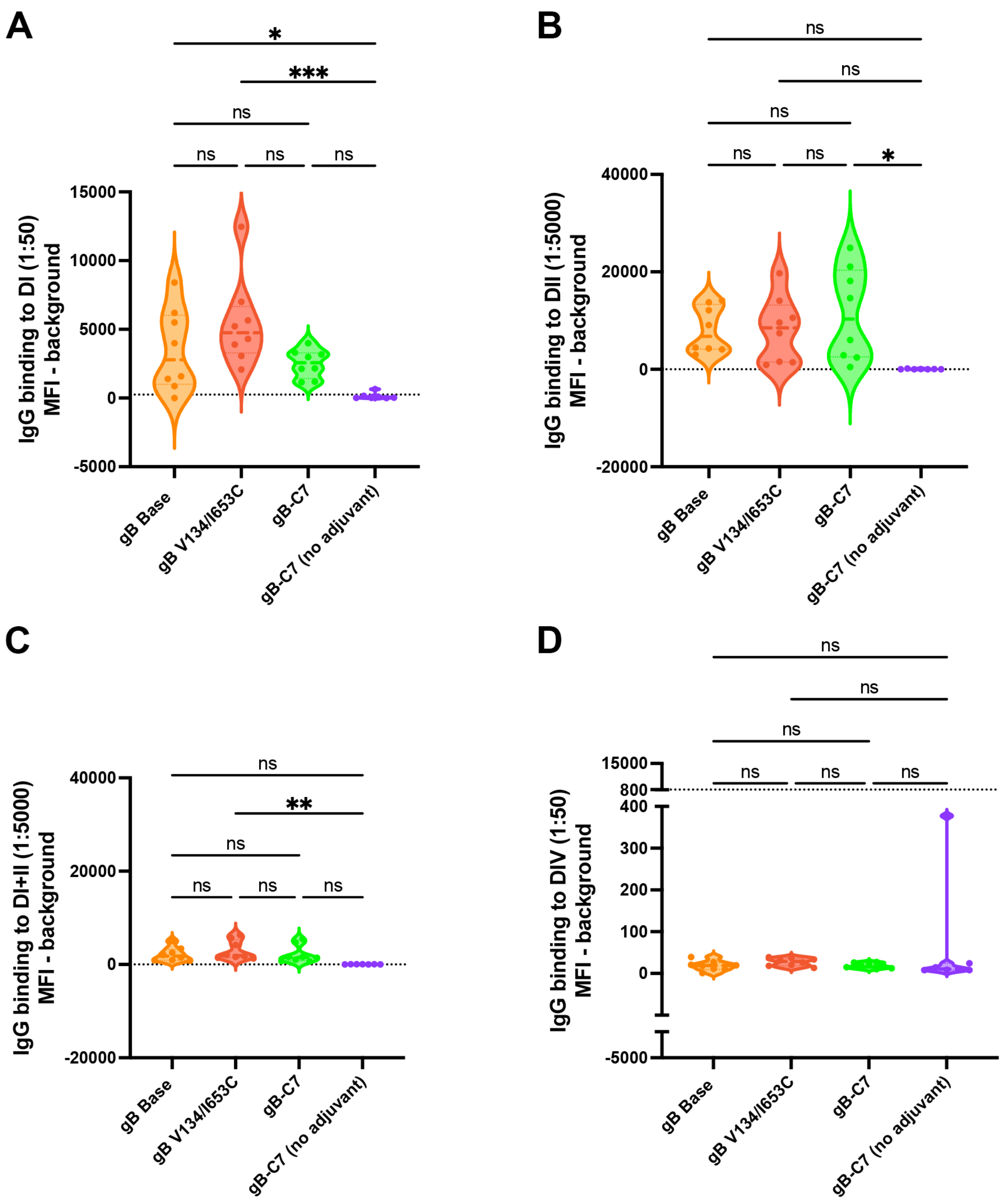

Figure S14. IgG mapping of immunized mouse sera. Sera from mice immunized with gB Base (orange), gB V134C/I653C (red), or gB-C7 (green), all adjuvanted with CpG 1018 plus alum or un-adjuvanted gB-C7 (purple), were assessed for binding to (**A**) structural domain I (DI), (**B**) DII, (**C**) DI+II, and (**D**) DIV of HCMV gB. Binding IgG antibody responses were measured using a Luminex-based binding antibody multiplex assay (BAMA) and reported as mean fluorescent intensity (MFI) with background binding subtracted. Plots represent averages from two independent experiments. In violin plots, horizontal lines represent the first quartile, median, and third quartile. Dotted line represents the average binding level of seronegative samples. Statistical significance was determined by one-way ANOVA followed by Tukey's HSD test in GraphPad Prism: * = p ≤ 0.05, ** = p ≤ 0.01, *** = p ≤ 0.001, **** = p ≤ 0.0001.

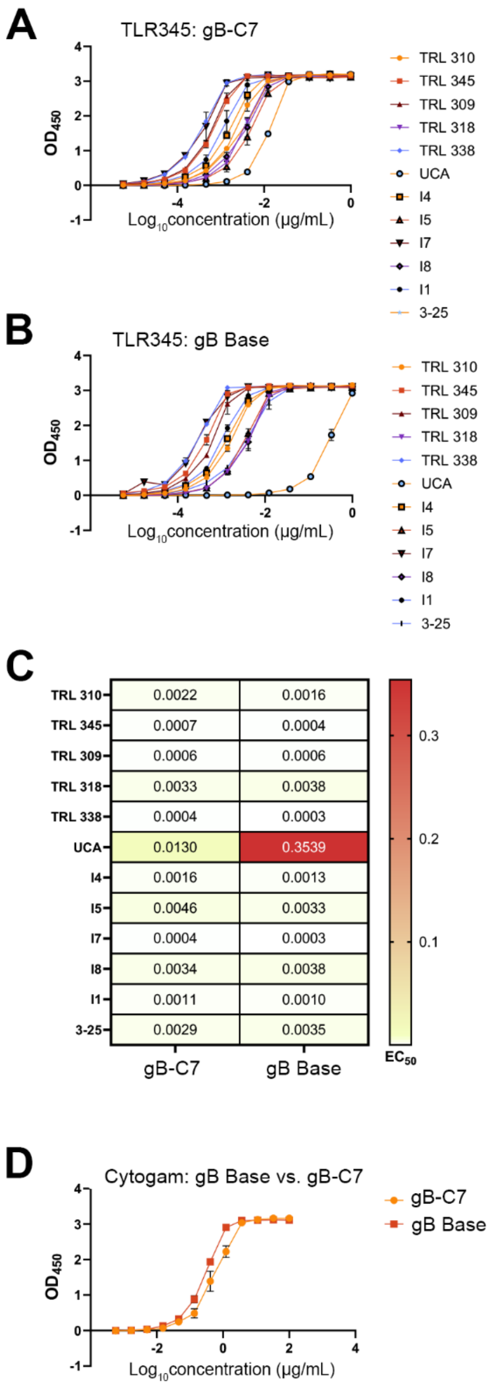

Figure S15. The AD-2S1-specific TRL345 Unmutated Common Ancestor binds gB-C7 more potently than gB Base. Binding of anti-gB AD-2 TRL345 lineage antibodies to (**A**) gB-C7 and (**B**) gB Base via ELISA. (**C**) ELISA plates were coated with gB Base or gB-C7 and incubated with the TRL345 lineage antibodies listed. Most of the TRL345 lineage antibodies exhibited similar binding to gB-C7 and gB Base. However, the TRL345 unmutated common ancestor (UCA) demonstrated higher binding to gB-C7 than to gB Base. (**D**) Cytogam was used as a positive control for binding gB-C7 and gB Base.

Table S1. Cryo-EM data collection and refinement statistics

Sample Composition	Postfusion gB Base in complex with 1G2 and 7H3 Fabs	Prefusion gB-C7 in complex with 1G2 and 7H3 Fabs	
**CRYO-EM DATA COLLECTION**			
Microscope (FEI)	Titan Krios	Titan Krios	
Voltage (kV)	300	300	
Detector	K3	K3	
Pixel size (Å/pix)	0.8332	0.8332	
Exposure ($e^-/Å^2$)	80	80	
Defocus range (µm)	1.0-3.0	1.0-2.5	
Micrographs collected at 0° tilt	1,308	12,524	
Micrographs collected at 30° tilt	7,371	0	
Micrographs used (all tilt angles)	7,939	11,558	
Total particle picks extracted	4,317,580	3,590,995	
Automation software	SerialEM 3.9.0 beta	SerialEM 3.9.0 beta	
**CRYO-EM REFINEMENT STATISTICS**			
Refinement	Global	Global	Local
Particles	211,132	165,982	497,946
Symmetry	C1	C3	C1
Map sharpening B-factor	-98	-86	-102
Resolution at FSC...			
Unmasked: 0.5 (Å)	6.0	3.8	4.6
Masked: 0.5 (Å)	3.8	3.2	3.7
Unmasked: 0.143 (Å)	4.0	3.3	3.9
Masked: 0.143 (Å)	3.4	2.8	3.1
**MODEL REFINEMENT AND VALIDATION STATISTICS**			
Composition			
Amino Acids (#)	2218	2958	
Ligands (Type: #)	BMA: 3, NAG: 42	BMA: 3, NAG: 30	
RMSD Bonds			
Length [Å] (# > 4s)	0.002 (0)	0.004 (0)	
Angles [°] (# > 4s)	0.44 (0)	0.57 (0)	
Ramachandran plot			
Outliers (%)	0.1	0.0	
Allowed (%)	3.8	5.0	
Favored (%)	96.1	95.0	
Rotamer outliers (%)	0.7	0.0	
Cβ outliers (%)	0.0	0.0	
CaBLAM outliers (%)	2.0	1.6	
CC (mask)	0.87	0.87	
MolProbity score	1.6	1.5	
Clash score	6.3	3.3	
Q-score	0.48	0.55	
PDB ID	8VYM	8VYN	
EMDB ID(s)	EMD-43667	EMD- 43670, 43671, 43672	

Table S2. Area under the curve from SEC traces for HCMV gB single substitution variants normalized to gB Base.

Substitution(s)	Normalized AUC_{HMW}	Normalized AUC_{LMW}	Normalized $AUC_{HMW+LMW}$	Normalized $AUC_{LMW/HMW}$
Q98C/G271C	0.14	0.46	0.20	3.35
Q98C/N658C	0.76	3.32	1.26	4.35
V134C/I653C	1.26	3.63	1.72	2.88
N220C/E657C	0.59	4.52	1.36	7.61
H222C/E657C	1.35	5.07	2.08	3.77
I356C/A500C	0.46	0.68	0.50	1.49
S367C/A503C	1.61	4.55	2.18	2.83
Q612C/R662C	0.57	1.42	0.74	2.47
S674C/E698C	1.82	2.98	2.04	1.64
V273K	1.61	1.66	1.62	1.03
D509A	0.38	0.51	0.40	1.36
D509L	0.05	0.11	0.06	2.30
S526K	0.94	1.30	1.01	1.38
D679V/E682N	1.84	2.29	1.96	1.24
Q98L/N658I	0.98	2.35	1.25	2.39
T100L/A267I	1.44	5.00	2.06	3.47
K130Y	1.75	4.02	2.33	2.29
K260W	1.11	2.53	1.36	2.27
L664F	1.54	1.92	1.61	1.25
N341P	2.03	1.53	1.94	0.75
L484P	1.72	4.09	2.14	2.38
V645P	0.95	2.03	1.17	2.14
D646P	0.98	1.81	1.12	1.85
E686P	1.78	1.25	1.69	0.70

AUC was quantified from SEC traces of purified gB single substitution variants. AUCs of the high molecular weight (HMW) peak (AUC_{HMW}), the low molecular weight (LMW) peak (AUC_{LMW}), and the sum of the two peaks ($AUC_{HMW+LMW}$) were normalized to respective gB Base AUCs expressed and purified within the same batch. The ratio of the normalized AUC_{LMW} relative to the normalized AUC_{HMW} ($AUC_{LMW/HMW}$) is reported at the far right.

Table S3. Anti-gB antibody titers and neutralization capacity of immunized mouse serum

Immunogen	SM5-1 Equivalent Titer (µg/mL)		Neutralization 1/ID_{50}	
	gB Base Binding	gB-C7 Binding	No complement	With complement
gB Base	1502.3	287.4	0.1	978.5
	1155.4	335.3	ND	492.4
	918.8	143.7	ND	1192.9
	1261.6	271.4	ND	635.7
	2152.3	370.3	133.4	659.6
	2352.2	222.6	ND	1300.2
	1729.8	259.8	193.7	2092.5
	902.0	134.0	ND	624.2
gB V134C/I653C	1703.4	374.8	ND	93.8
	896.8	179.2	ND	362.8
	1771.6	260.9	ND	425.5
	1461.3	414.9	ND	670.7
	2097.1	209.3	0.5	197.8
	738.3	106.6	ND	398.7
	2154.9	399.8	ND	415.3
	797.1	90.3	ND	25.4
gB-C7	1000.5	229.4	ND	108.8
	805.6	129.8	ND	59.2
	1803.9	297.6	4.3	124.0
	976.0	224.3	ND	200.2
	1887.4	338.5	ND	133.5
	1533.0	231.6	ND	169.5
	804.4	133.6	2.7	9.0
	912.8	200.2	ND	13.5
gB-C7 (no adjuvant)	142.7	26.1	ND	ND
	35.1	5.3	ND	ND
	40.9	8.4	ND	0.7
	91.8	20.0	ND	ND
	32.9	2.0	ND	ND
	378.5	41.0	ND	ND
	64.2	18.3	ND	ND
	61.4	14.5	ND	0.5

Antibody binding titers against gB Base and gB-C7 of immunized mouse sera. Titers were measured by ELISA relative to SM5-1 mFc standard. The capacity of immunized mouse sera to neutralize AD169 infection of MRC-5 fibroblasts is reported as 1/ID_{50}. Data is reported as an average of two independent experiments. ND: Not detected.

SI References

1. Y. Liu *et al.*, Prefusion structure of human cytomegalovirus glycoprotein B and structural basis for membrane fusion. *Sci Adv* **7** (2021).

2. S. Chandramouli *et al.*, Structure of HCMV glycoprotein B in the postfusion conformation bound to a neutralizing human antibody. *Nat Commun* **6**, 8176 (2015).

3. H. G. Burke, E. E. Heldwein, Crystal Structure of the Human Cytomegalovirus Glycoprotein B. *PLoS Pathog* **11**, e1005227 (2015).

4. J. Jumper *et al.*, Highly accurate protein structure prediction with AlphaFold. *Nature* **596**, 583-589 (2021).
